# Glycine receptor autoantibody binding to the extracellular domain is independent from receptor glycosylation

**DOI:** 10.3389/fnmol.2023.1089101

**Published:** 2023-02-13

**Authors:** Vera Rauschenberger, Inken Piro, Vikram Babu Kasaragod, Verena Hörlin, Anna-Lena Eckes, Christoph J. Kluck, Hermann Schindelin, Hans-Michael Meinck, Jonathan Wickel, Christian Geis, Erdem Tüzün, Kathrin Doppler, Claudia Sommer, Carmen Villmann

**Affiliations:** ^1^Institute of Clinical Neurobiology, University Hospital, Julius-Maximilians-University of Würzburg, Würzburg, Germany; ^2^Department of Neurology, University Hospital Würzburg, Würzburg, Germany; ^3^Rudolf Virchow Centre for Integrative and Translational Bioimaging, Julius-Maximilians-University of Würzburg, Würzburg, Germany; ^4^Institute of Biochemistry, Emil-Fischer-Center, FAU Erlangen-Nürnberg, Erlangen, Germany; ^5^Department of Neurology, University Hospital Heidelberg, Heidelberg, Germany; ^6^Section Translational Neuroimmunology, Department of Neurology, Jena University Hospital, Jena, Germany; ^7^Institute of Experimental Medicine, Istanbul University, Istanbul, Türkiye

**Keywords:** glycine receptor, autoantibodies, glycosylation, extracellular domain, adsorption

## Abstract

Glycine receptor (GlyR) autoantibodies are associated with stiff-person syndrome and the life-threatening progressive encephalomyelitis with rigidity and myoclonus in children and adults. Patient histories show variability in symptoms and responses to therapeutic treatments. A better understanding of the autoantibody pathology is required to develop improved therapeutic strategies. So far, the underlying molecular pathomechanisms include enhanced receptor internalization and direct receptor blocking altering GlyR function. A common epitope of autoantibodies against the GlyRα1 has been previously defined to residues ^1^A-^33^G at the N-terminus of the mature GlyR extracellular domain. However, if other autoantibody binding sites exist or additional GlyR residues are involved in autoantibody binding is yet unknown. The present study investigates the importance of receptor glycosylation for binding of anti-GlyR autoantibodies. The glycine receptor α1 harbors only one glycosylation site at the amino acid residue asparagine 38 localized in close vicinity to the identified common autoantibody epitope. First, non-glycosylated GlyRs were characterized using protein biochemical approaches as well as electrophysiological recordings and molecular modeling. Molecular modeling of non*-*glycosylated GlyRα1 did not show major structural alterations. Moreover, non-glycosylation of the GlyRα1^N38Q^ did not prevent the receptor from surface expression. At the functional level, the non-glycosylated GlyR demonstrated reduced glycine potency, but patient GlyR autoantibodies still bound to the surface-expressed non-glycosylated receptor protein in living cells. Efficient adsorption of GlyR autoantibodies from patient samples was possible by binding to native glycosylated and non-glycosylated GlyRα1 expressed in living not fixed transfected HEK293 cells. Binding of patient-derived GlyR autoantibodies to the non-glycosylated GlyRα1 offered the possibility to use purified non-glycosylated GlyR extracellular domain constructs coated on ELISA plates and use them as a fast screening readout for the presence of GlyR autoantibodies in patient serum samples. Following successful adsorption of patient autoantibodies by GlyR ECDs, binding to primary motoneurons and transfected cells was absent. Our results indicate that the glycine receptor autoantibody binding is independent of the receptor’s glycosylation state. Purified non-glycosylated receptor domains harbouring the autoantibody epitope thus provide, an additional reliable experimental tool besides binding to native receptors in cell-based assays for detection of autoantibody presence in patient sera.

## Introduction

Glycine receptor (GlyR) autoantibodies have been detected in patients suffering from stiff-person syndrome (SPS) or progressive encephalomyelitis with rigidity and myoclonus (PERM) (Carvajal-Gonzalez et al., 2014; Martinez-Hernandez et al., 2016; Dalmau et al., 2017). SPS is characterized by stiffness and painful spasms in muscles of the lower trunk and legs, while PERM is a more complex form with additional sensory disturbance, brainstem dysfunctions, epilepsy, ataxia and/or dysautonomia (Dalmau et al., 2017). The targeted protein of GlyR autoantibodies is an inhibitory chloride-permeable ion channel which belongs to the Cys-loop receptor family (Lynch, 2004). GlyRs form pentameric receptor channels composed of α (α1-3) and β subunits in α-homomeric or αβ-heteromeric configurations (Patrizio et al., 2017; Kasaragod and Schindelin, 2019;Yu et al., 2021; Zhu and Gouaux, 2021). The favored receptor stoichiometry of heteromeric GlyRs has been suggested with a 4α:1β ratio (Yu et al., 2021; Zhu and Gouaux, 2021). During GlyR maturation and trafficking to the cellular membrane, GlyRs are N-glycosylated (Griffon et al., 1999). In contrast to other α or the β subunit, the GlyRα1 subunit carries a single glycosylation site at the asparagine residue 38 of the mature protein (Pult et al., 2011; Schaefer et al., 2018). The glycosylation site is located in close neighborhood to a recently identified common binding epitope (^1^A-^33^G; mature protein) of patient-derived GlyR autoantibodies in the N-terminus of the receptor (Rauschenberger et al., 2020).

Upon binding, GlyR autoantibodies are able to activate the complement system, crosslink receptors, and lead to receptor internalization with subsequent degradation (Carvajal-Gonzalez et al., 2014). Recent analyses showed direct antibody effects on the extracellular receptor domain (ECD) impairing GlyR function and thus reducing inhibitory neurotransmission (Carvajal-Gonzalez et al., 2014; Crisp et al., 2019; Rauschenberger et al., 2020). Commonly, SPS patients harboring GlyR autoantibodies are treated with immunotherapies such as intravenously applied polyvalent immunoglobulin (IVIG) or plasmapheresis, and further symptomatic immunotherapy by enhancing GABAergic inhibition, e.g., by benzodiazepines (Howard, 1963; Vicari et al., 1989; Pagano et al., 2014). However, therapeutic responses are often limited and relapses occur which interfere with patient recovery (Hutchinson et al., 2008; Damasio et al., 2013; McKeon et al., 2013; Carvajal-Gonzalez et al., 2014; Doppler et al., 2016; Dalmau et al., 2017). Even though patient sera analysis for the presence of autoantibodies provides sometimes false negative results if not tested on living cells expressing native proteins (Vincent et al., 2012). Cell-based assays, however, are subject to variations between independent experiments and are rather time-consuming. The use of high-throughput peptide arrays for epitope screening of patient autoantibody binding has also been demonstrated to represent another experimental tool to investigate autoantibody binding (Talucci and Maric, 2023). In case of conformational epitopes, however, there are also limitations with such approaches. Recently, binding patterns of patient-derived monoclonal antibodies have been studied using purified full-length receptors or domains in distinct conformations bound to ELISA plates followed by structural analysis, e.g., single particle cryo-EM of combined receptor and autoantibody proteins and functional analyses (Chou et al., 2022; Tajima et al., 2022).

Here, we investigated first the role of receptor glycosylation for GlyR autoantibody binding followed by the use of purified GlyR ECDs to study the binding pattern of patient-derived serum samples. Our molecular modeling analysis argued for no large structural alterations of the receptor in its non-glycosylated form. Non-glycosylated GlyRα1 receptors were characterized for alterations in surface expression level, functional consequences due to lack of glycosylation as well as for patient autoantibody binding. We identified that purified GlyRα1 and GlyRα2 ECDs efficiently adsorb patient autoantibodies from patient samples.

## Materials and methods

### Ethical statement

#### Human

Permission for experiments with patient material has been issued by the Ethics Committee of the Medical Faculty of the University of Würzburg, Germany with the project on “Autoantibodies and glycinergic dysfunction - pathophysiology of associated motor disorders” (#2019042402). All participants gave written informed consent to take part in the study.

#### Animals

CD1 (strain number 022; Charles River, Wilmingthon, MA, United States) pregnant female mice were used to isolate spinal cord neuronal cultures at the embryonic stage 12.5 (E12.5). Experiments were approved by the local veterinary authority (Veterinäramt der Stadt Würzburg, Germany) and the Ethics Committee of Animal Experiments, i.e., Regierung von Unterfranken, Würzburg, Germany (license no.: FBVVL 568/200-324/13).

### Patients

Serum from five patients (Pat1-5) with anti-GlyR autoantibodies was used for the experiments. Clinical data of two patients (Pat1 with SPS, Pat5 with PERM) were described previously (Rauschenberger et al., 2020). Serum from five healthy individuals served as negative control (HC), a serum from a patient with multiple sclerosis was used as disease control (DC).

Patients 2 and 3 did not have a history of cancer, SPS or PERM but another neurological disorder. Their serum and CSF samples did not exhibit antibodies directed against other neuronal surface antigens (NMDAR, AMPAR, LGI1, CASPR2, GABA_A_R, GABA_B_R) but autoantibodies against the GlyR assessed by appropriate cell-based assays (Ekizoglu et al., 2014). Sera of patients 2 and 3 were included in the present study to examine general molecular mechanisms for autoantibody binding and detection.

*Patient 2* was a 33-year old woman with a 16-year history of focal epilepsy of unknown cause, characterized with focal temporal seizures evolving to bilateral convulsive seizures and loss of consciousness. Her CSF and MRI examinations were normal. She favorably responded to antiepileptic medications (oxcarbazepine and topiramate) and thus immunotherapy was not considered.

*Patient 3* was a 14-year old girl with a 6-year history of unclassified epileptic encephalopathy characterized with generalized tonic–clonic seizures and atypical absences. She showed mildly progressive cognitive decline with normal metabolic screening, normal CSF findings and mild cerebral atrophy on MRI. EEG showed generalized discharges and focal spikes over the right frontotemporal region. She favorable responded to IVIG treatment and anti-epileptic medications (carbamazepine and valproate).

*Patient 4*, a 71-year old male at blood withdrawal, developed symptoms of SPS (PERM) at age 65. He suffered from stiffness and spasms of the right arm, falls with bone fractures and a pronounced startle reaction, and was finally wheelchair bound. Extensive search for a malignoma and for other autoantibodies than GlyR antibodies was negative. Under i.v. steroid pulse therapy every 4 months and oral clonazepam at a dose of 0.25 mg in the morning and 0.375 mg in the evening he recovered the ability to walk, and his condition remained mostly stable.

### Site-directed mutagenesis of the GlyRα1 variant

Site-directed mutagenesis was used to create the non-glycosylation mutant GlyRα1^N38Q^. The human GlyRα1 cDNA in pRK5 vector (gift †P. Seeburg) was used as template. Sequence correctness was verified (Eurofins Genomics Germany GmbH, Ebersberg, Germany). For transfection with GFP, the eGFP-N1 plasmid (Takara Bio Europe, Saint-Germain-en-Laye, France) was used.

### Cell lines

HEK293 cells (human embryonic kidney, ATCC^®^ CRL-1573™, Wesel, Germany) were cultured in Minimum Essential Media (MEM, Life Technologies, Darmstadt, Germany) supplemented with 10% fetal calf serum (FCS), glutaMAX (200 mM), sodium pyruvate (100 mM), penicillin (10,000 U/ml), and streptomycin (10,000 μg/ml). The cells were incubated at 37°C and 5% CO_2_.

### Transfection of cells

HEK293 cells were transfected using a calcium phosphate precipitation method. Plasmid DNA (1 μg/μl; 1 μg of each plasmid was used in cotransfections of GFP and GlyRα1 variants or the empty pRK5 vector (MOCK control) for 3 cm dishes containing four coverslips and 180,000 cells seeded for immunocytochemical experiments and electrophysiological recordings; transfections in 10 cm dishes containing 1.8 × 10^6^ HEK293 cells for biotinylation experiments used 10 μg of plasmid per dish), 2.5 M CaCl_2_, 0.1× TE buffer (Tris/EDTA) and 2× HBS (50 mM HEPES, 12 mM glucose, 10 mM KCl, 280 mM NaCl, 1.5 mM Na_2_HPO_4_) were incubated for 20 min at 20°C. The mixture was applied to the cells. Cells were washed after 5–6 h and used for experiments after 24–72 h.

### Cultivation of primary spinal cord neurons

Pregnant CD1 mice were euthanized with CO_2_ and cervical dislocation. Spinal cords were extracted from embryonic day 12.5 (E12.5) embryos. Spinal cord tissue fragments were triturated three times. Supernatants with cells were centrifuged at 400 rpm for 20 min. Cells were counted and plated on poly-D-lysine-covered coverslips using a density of 150,000 cells/3 cm dish. Neurons were grown in Neurobasal medium (Life Technologies, Darmstadt, Germany) containing 5 ml of L-glutamine (200 mM) and B27 supplement (Life Technologies, Darmstadt, Germany) with an exchange of 50% medium after 7 days in culture. Neurons were used for experiments at day *in vitro* 14 (DIV14).

### Immunocytochemical stainings

#### Live staining

Transfected HEK293 cells were incubated with patient (Pat) serum (1:50 in MEM medium with supplements), GlyRα1 specific antibody MAb2b (1:500; 146,111, RRID:AB_2278673, Synaptic Systems, Göttingen, Germany), or GlyR pan-α antibody MAb4a (1:500; 146,011, RRID:AB_887722, Synaptic Systems, Göttingen, Germany) for 2 h on ice. After three washing steps in phosphate-buffered saline (PBS, pH 7.4), cells were fixed for 20 min with 4% paraformaldehyde/4% sucrose on ice. A blocking step using 5% goat serum (PAA Laboratories, Cölbe, Germany) was applied for 30 min at 20°C. Cells were incubated for 30 min with secondary antibodies goat anti-human Cy3 (1:500; 109–165-003, RRID:AB_2337718, Dianova, Hamburg, Germany), goat anti-mouse Cy3 (1:500; 115–165-003, RRID:AB_2338680, Dianova, Hamburg, Germany) or goat anti-rabbit Alexa 488 (1:500, 111–545-003, RRID:AB_2338046, Jackson ImmunoResearch Laboratories Inc., West Grove, United States). Cells were incubated with 4′,6-diamidino-2-phenylindole (DAPI, 1:5,000; Thermo Fisher Scientific, Waltham, MA, United States) for 5 min at 20°C. Cells were mounted using mowiol (Sigma Aldrich, Munich, Germany). In case permeabilization was required for antibody binding, 0.2% Triton-X-100 in blocking solution was used for 30 min at 20°C. Motoneurons have been fixed and permeabilized before staining with the synapsin antibody (1:500, 574,778, RRID:AB_2200121, Merck, Darmstadt, Germany) as well as the pre-adsorbed supernatants containing patient serum following binding to GlyRα1 ECD.

#### Adsorption

Transfected HEK293 cells (3 cm dish with 4 cover slips; 200,000 cells) were incubated with Pat serum (1:50 in MEM medium with supplements) or GlyRα1 antibody MAb2b (1:500) for 1 h at 20°C. The supernatant containing so far unbound antibodies was transferred to the next coverslip with transfected HEK293 cells and again incubated for 1 h at 20°C. In total, three transfers were performed.

An Olympus microscope (Fluoview ix1000, Olympus, Hamburg, Germany) was used to take images of stained cells. Further image processing was performed using the software Fiji (Schindelin et al., 2015).

### Structural analysis of GlyR wildtype and the mutant N38Q

The crystal structure of human glycine receptor α3 homopentamer in complex with strychnine (PDB: 5CFB) (Huang et al., 2015) was used as template to introduce the mutation and to analyse possible structural rearrangements or interaction changes in the surrounding region of the mutation. *In silico* site-directed mutagenesis (N38Q) was carried out in Coot taking basic geometry of the mutated residue and clashes of the surrounding residues into account (Emsley and Cowtan, 2004). The figures were prepared by using PyMOL^1^.

### Cell lysates and de-glycosylation

Cell lysates of transfected HEK293 cells were prepared using the CytoBuster Protein Extraction Reagent (Merck Millipore, Billerica, MA, United States). To verify GlyR N-glycosylation, lysates of GlyRα1^WT^ and GlyRα1^N38Q^ were incubated with peptide N-glycosidase F (PNGase F) or endoglycosidase H (EndoH) according to manufacturer’s instructions (P0704S and P0702S, New England BioLabs, Ipswich, MA, United States).

### Biotinylation assay

Biotinylation experiments were performed as described previously (Atak et al., 2015).

### Western blot and immunostaining

40 μg protein/lane were loaded on a 11% polyacrylamide gel. Proteins were transferred to nitrocellulose membrane (GE HealthCare, Freiburg, Germany). Membranes were washed in TBST (TBS with 1% Tween 20), blocked for 1 h in 3% bovine serum albumin (BSA, Carl Roth, Karlsruhe, Germany) and incubated in MAb4a (1:500; 146,011, RRID:AB_887722, Synaptic Systems, Göttingen, Germany), anti-gephyrin (1:1,000; 147,111, RRID:AB_887719, Synaptic Systems, Göttingen, Germany), anti-GFP (1:5,000; SC8334, RRID:AB_641123, Santa Cruz Biotechnology, Dallas, TX, United States) or anti-pan-cadherin antibody (1:1000; 4,068, RRID:AB_2158565, Cell Signaling, Danvers, MA, United States) overnight at 4°C. As secondary antibodies horse radish peroxidase (HRP) conjugated goat anti-mouse (1:15,000, 115–035-146, RRID:AB_2307392, Dianova, Hamburg, Germany) or goat anti-rabbit secondary antibodies (1:15,000, 111–036-003, RRID:AB_2337942, Dianova, Hamburg, Germany) were used. Signal detection was done using the SuperSignal West (Thermo Fisher Scientific, Waltham, MA, United States). Western Blot Images were taken using the Chemostar Touch Imager (Intas Science Imaging Instruments, Göttingen, Germany).

### Electrophysiological recordings

Electrophysiological recordings using the patch-clamp method in a whole-cell configuration were performed. Transfected HEK293 cells were used for recordings at room temperature 21°C. Used borosilicate capillaries had open resistances of 3.5–5.5 MΩ and were filled with internal buffer (120 mM CsCl, 20 mM N(Et)_4_Cl, 1 mM CaCl_2_, 2 mM MgCl_2_, 11 mM EGTA, 10 mM HEPES; pH 7.2, adjusted with CsOH). The external buffer contained (137 mM NaCl, 5.4 mM KCl, 1.8 mM CaCl_2_, 1 mM MgCl_2_, 5 mM HEPES; pH 7.35, adjusted with NaOH). For determination of maximal current amplitudes (I_max_) and EC_50_ values, glycine was used in a concentration series of 10 μM, 30 μM, 60 μM, 100 μM, 300 μM, 600 μM, 1 mM glycine The agonist was applied using the Octaflow II system (ALA Scientific Instruments, Farmingdale, NY, United States). Current responses were amplified with an EPC-10 amplifier (HEKA, Elektronik GmbH, Lambrecht/Pfalz, Germany) and measured at a holding potential of −60 mV using Patchmaster Next software (HEKA Elektronik GmbH, Lambrecht/Pfalz, Germany). The analysis included cells with input resistances above 100 MΩ-1 GΩ, leak currents smaller than −300 pA and capacitances of 11–13 pF. Recorded maximal currents were plotted and fitted using the hill 1 function in Origin 2019 (Originlab Corporation, Northampton, United States) to examine EC_50_ values.

### ECD preparation

Lysis, preparation of the inclusion bodies (IB), refolding and ECD purification were performed according to the established protocol by Breitinger et al. with some minor changes (Breitinger et al., 2004). In brief, GlyR ECDs (GlyRα1 and GlyRα2; both in the vector pET30) were expressed in *Escherichia coli* as IBs. After lysis, the lysates were centrifugated (20,000 g, 4°C, 30 min). The pellets, including the IBs, were washed twice with Tris/HCl-buffer, pH 7.4, 0.5% Triton-X-100. A third washing step was done with a Tris/HCl buffer containing 1 M urea instead of Triton-X-100. The IB-pellet was dissolved in 8 M urea in 100 mM Na_2_PO_4_, 10 mM Tris/HCl pH 8. Preparative refolding was done by dialyzing 10 ml (1 mg/ml) IB-solution against a volume of 5 l buffer with decreasing concentrations of urea, starting from 5 M urea in 50 mM Tris/HCl pH 8 in nine subsequent steps, each lasting at least 4 h at 4°C. The last dialysis buffer contained 5 mM Na-phosphate, pH 7.4 and 150 mM NaCl. To get rid of aggregated and non-refolded ECDs, the preparation was ultracentrifugated for 40 min at 100,000 g, 4°C. To exclude aggregated proteins further, a size exclusion chromatography using a Superose column was performed. The purity and size of the refolded GlyRαl or α2 ECDs was verified on 11% PAA gels and stained using Coomassie solution (0.1% Coomassie brilliant blue R250, 25% methanol, 7.5% acetic acid). De-staining was performed until protein bands became visible (40% Methanol, 10% acetic acid). GlyRα2 ECD refolding was proven by Circular-dichroism (CD) spectroscopy.

### CD spectroscopy

CD spectroscopy was conducted with a Jasco J-810 spectropolarimeter. Far-UV spectra from 190 to 270 nm were recorded at a scanning speed of 50 nm/min with a response time of 1 s and a bandwidth of 1.5 nm. Spectra were baseline corrected by subtracting buffer runs. Ten individual scans were taken and averaged. The buffer was exchanged to 50 mM N-phosphate buffer, pH 8 with ultrafiltration units (Sartorius Vivaspin 500, Göttingen).

### ELISA

ELISA plates (Sarstedt, Nümbrecht, Germany) were coated overnight with 2 μg/ml purified and refolded GlyRαl or α2 ECD. ELISA plates were washed 5x with H_2_O_dest._ and blocked with 10% BSA diluted in PBS containing 0.05% Tween 20 for 1 h at 37°C. Wells were incubated with patient serum (1:100 in blocking solution) or GlyR pan-α antibody MAb4a (1:500) for 1 h at 37°C. The supernatants were transferred three times into fresh coated wells subsequent to incubation with GlyRα1/α2 and GFP co-transfected HEK293 cells or primary spinal cord neurons. The ELISA plates were further processed with washing steps in PBS and secondary antibody goat anti-mouse (1:20,000 in blocking, 115–035-146, RRID:AB_2307392, Dianova, Hamburg, Germany) or goat anti-human HRP (1:20,000 in blocking, 109–035-088, RRID:AB_2337584, Dianova, Hamburg, Germany) incubation for 1 h at 37°C. TMB solution (00–4,201–56, Thermo Fisher Scientific, Waltham, MA, United States) was added and reaction was stopped after 15 min with 1 M H_3_PO_4_. Absorbance was read with a Wallac 1,420 Victor2 Microplate Reader (Perkin Elmer, Waltham, MA, United States) at 450 nm. As controls, uncoated ELISA plates have been used with the same experimental procedure to test for specificity. Absorbance data upon binding to plates without the target protein have been subtracted from values exhibited from ELISA plates with GlyR ECDs bound.

### Statistical analysis

Data sets were tested for statistical significance by using unpaired *t*-test with the Holm-Šidák correction for multiple samples or the one-way ANOVA with Šidák’s multiple comparison *post hoc* test: *value of *p*<0.05; **value of *p*<0.01; ***value of *p*<0.001, ****p < 0.0001. Bar diagrams indicate mean values ± standard error of the mean (SEM) or as noted otherwise. Statistical analyses have been performed using GraphPad Prism version 9.3.1.

## Results

### De-glycosylated GlyRα1 shows no major structural alteration

**Figure 1:**
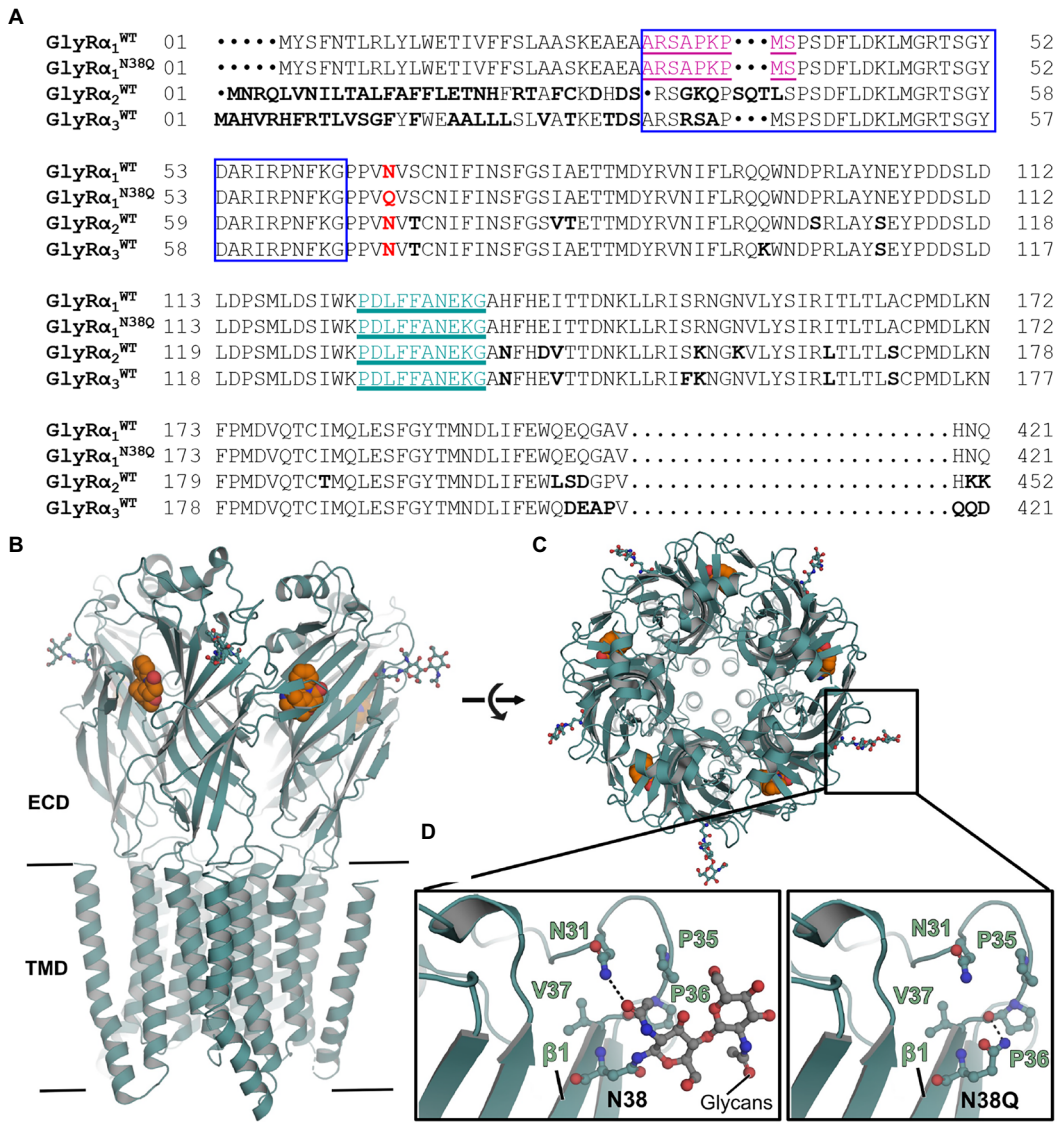
Non-glycosylated GlyRs show no obvious structural alterations. **(A)** Alignment of GlyRα1^WT^, GlyRα1^N38Q^, GlyRα2^WT^, and GlyRα3^WT^. The position of the amino acid exchange of the non-glycosylation mutant (N38Q) is indicated in red. All GlyRα subunits share the asparagine at this position and with this a consensus site for glycosylation N-X-S/T. The MAb2b antibody epitope is depicted in purple underlined (Ala1-Ser9, numbers refer to mature protein) and the MAb4a antibody epitope is shown in cyan underlined (Pro96-Gly105). The autoantibody epitope (Ala29-Gly62) (Rauschenberger et al., 2020) is indicated by a blue box. Non-homologous amino acids between GlyRα1^WT^, α2^WT^ and α3^WT^ are highlighted as bold letters. **(B)** Crystal structure of human GlyRα3 homopentamer (PDB: 5CFB) (Huang et al., 2015) in side view and top view **(C)**. The antagonist strychnine (orange) is shown in space-filling representation and glycans attached to N38 in ball and stick representation (marked by black box). **(D)** Enlarged views at position Asn38 in GlyRα3^WT^ (left) and GlyRα3^N38Q^ (right). The proline residue lost a hydrogen bond to asparagine Asn31, but no further obvious changes were detected.

The proposed common epitope of GlyR autoantibodies between amino acid residues 29–62 (refers to residues 1–33 in the mature protein) (Rauschenberger et al., 2020) is close to the GlyR glycosylation site (Asn38, number refers to mature GlyR protein, Figure 1A). The region surrounding the glycosylation site might be important for autoantibody binding as it has been shown for other autoantibody subtypes (Gleichman et al., 2012; Castillo-Gomez et al., 2017). The N-glycosylation position 38 of the mature GlyRα1 is conserved in GlyRα2 and GlyRα3 (Figure 1A). First, we investigated the impact of the mutation on surrounding interactions using *in silico* mutational analysis. The crystal structure of human GlyRα3 homomer in complex with strychnine was used as template as it displays the best resolution among available glycine receptor structures (Figure 1B; Huang et al., 2015). In the wildtype structure, *N*-acetyl-D-glucosamine covalently linked to Asn38 interacts with Asn31 residing in the loop connecting the first α-helix and the first β-strand *via* a hydrogen bond (Figures 1C,D). In the mutant version (N38Q), this interaction was lost, while generating a possible interaction between the side chain of N38 with the main chain carboxylate of the P36 (Figure 1D). However, the overall structure in this region is unchanged between the glycosylated and the non-glycosylated form (Figure 1D, compare left and right image).

### Non-glycosylation of the GlyR neither prevents surface expression nor autoantibody binding

**Figure 2:**
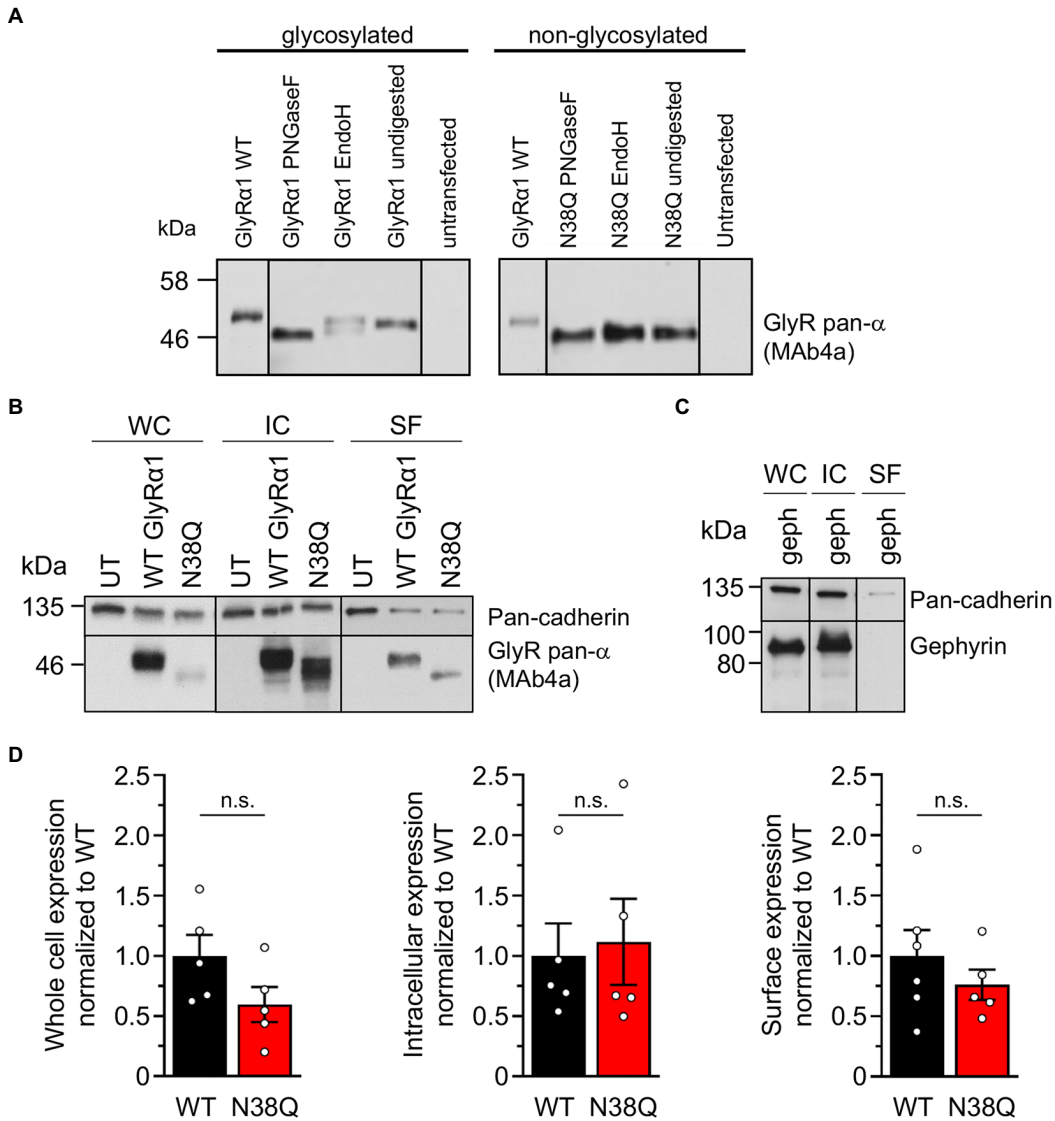
Lack of glycosylation in GlyRα1 leads to slightly decreased whole cell and cell surface expression level. **(A)** Cell lysates of GlyRα1^WT^ (left) and GlyRα1^N38Q^ (right) undigested or treated with glycosidases PNGaseF and EndoH. Blots were stained with MAb4a and lysates of untransfected HEK293 cells served as negative controls. **(B)** Representative blots of whole cell (WC), intracellular (IC) and surface (SF) protein levels of pan-cadherin and glycine receptor (MAb4a) of transfected HEK293 cells with GlyRα1^WT^, GlyRα1^N38Q^ compared to untransfected cells (UT). **(C)** Positive control blot of HEK293 cells transfected with gephyrin. **(D)** Quantification of whole cell, intracellular and surface expression of GlyRα1^WT^ (black), and GlyRα1^N38Q^ (red). Expression levels were normalized to pan-cadherin signal and GlyRα1^WT^ signal was set to 1 afterwards. Number of independent experiments *n* = 5–6; n.s., non-significant.

To verify the glycosylation state of the mutant GlyRα1^N38Q^, cell lysates of GlyRα1^WT^ and GlyRα1^N38Q^ transfected HEK293 cells were treated with the glycosidases PNGaseF and EndoH. In contrast to GlyRα1^WT^, PNGaseF and EndoH treated GlyRα1^N38Q^ showed no reduction in molecular weight compared to untreated samples (Figure 2A), thus demonstrating that the mutant GlyRα1^N38Q^ is present in a non-glycosylated isoform. The two bands observed after EndoH digestion of GlyRα1^WT^ result most probably from additional glycosylation modifications making the GlyR complex partially resistant to EndoH digestion (Bormann et al., 1993).

**Figure 3:**
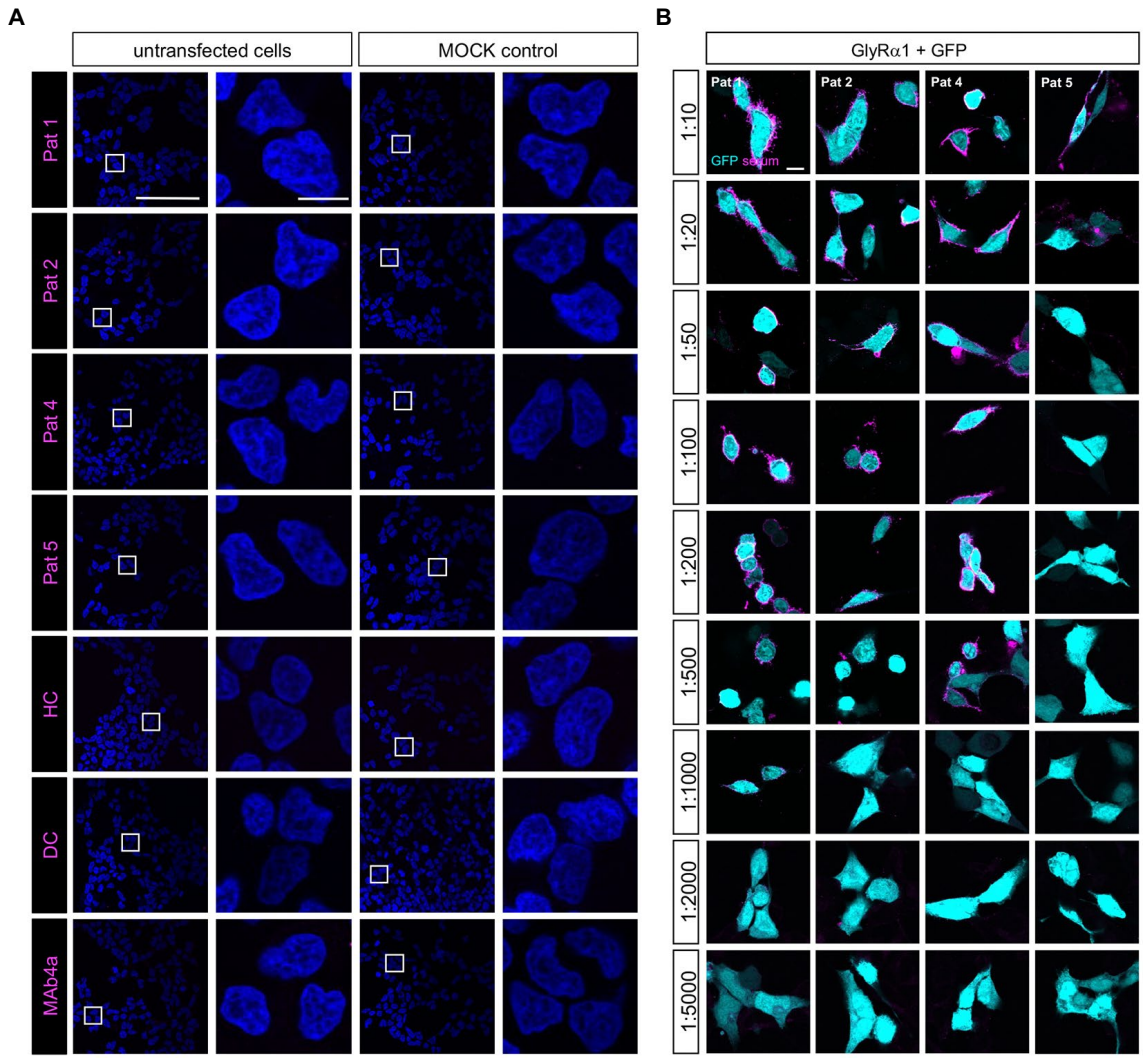
Patient sera bind to GlyRα1 transfected but not untransfected cells and differ in GlyR autoantibody titres. **(A)** Patient sera were first tested on untransfected HEK293 cells and cells transfected with the empty pRK5 plasmid. As negative controls, serum from a patient suffering from multiple sclerosis (disease control, DC) and a mix of sera from healthy controls (HC) were used. As control that untransfected cells do not endogenously express the GlyR, MAb4a (binds to all GlyRα subunits) was used. Scale bar for left columns (untransfected and MOCK) represents 100 μm, right columns 10 μm. **(B)** Patient sera were investigated in a dilution series from 1:10 to 1:5000 for binding to transfected HEK293 cells with GlyRα1 and GFP as internal transfection efficiency control. Incubation with sera was performed for 1 h at living cells, followed by fixation. As secondary antibody an anti-human Cy3 antibody was used (magenta). Pat3 was excluded from analysis due to limited amount of serum available. Scale bar represents 10 μm. All stainings were performed three times (*n* = 3) and representative images are shown.

**Figure 4:**
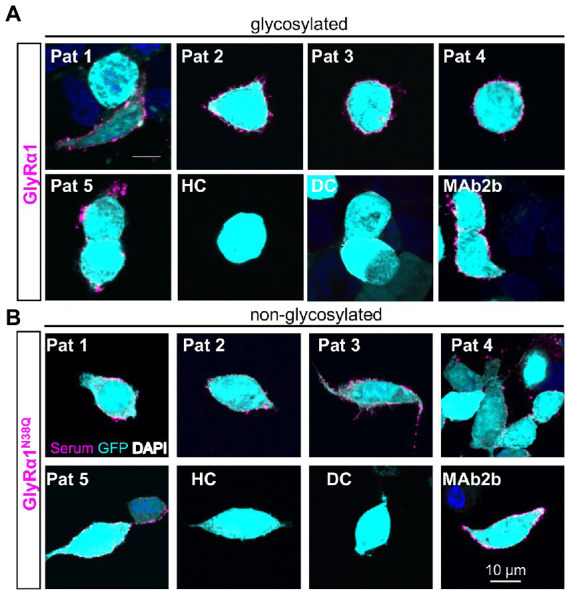
Patient anti-GlyR autoantibodies bind to the glycosylated and the non-glycosylated receptor. **(A)** HEK293 cells co-transfected with GlyRα1 and GFP (cyan, transfection control), followed by an immunocytochemical staining with patient sera (Pat1-5) or MAb2b antibody (magenta). As negative controls, serum from a patient suffering from multiple sclerosis (disease control, DC) and a mix of sera from healthy controls (HC) were used. **(B)** Immunocytochemical stainings of GlyRα1^N38Q^ and GFP (cyan) co-transfected HEK293 cells that were incubated with patient sera 1–5 or MAb2b (magenta). Healthy control (HC) and disease control (DC) were used as negative controls. Scale bar refers to 10 μm. Immunocytochemical stainings were performed in three independent experiments (*n* = 3).

To verify the amount of non-glycosylated GlyRα1^N38Q^ at the cellular surface, biotinylation assays were performed discriminating between whole cell, intracellular and surface protein fractions. Signal intensities were quantified relative to pan-cadherin signals and normalized to GlyRα1^WT^. Whole cell expression of GlyRα1^N38Q^ was reduced but not significantly changed compared to GlyRα1^WT^ (relative whole cell expression GlyRα1^WT^ 1.0 ± 0.16; GlyRα1^N38Q^ 0.57 ± 0.13; *p* = 0.1118). A similar decrease was observed for expressed GlyRα1^N38Q^ receptors at the cell surface (35% compared to GlyRα1^WT^; relative surface expression GlyRα1^WT^ 1.0 ± 0.2; GlyRα1^N38Q^ 0.65 ± 0.12; *p* = 0.3888; Figures 2B,D). Gephyrin-transfected cells were used as control. As expected for such an intracellular protein, gephyrin was not detectable in the surface fraction (Figure 2C). Hence, the GlyRα1^N38Q^ is expressed at the cell surface, a prerequisite to test for binding of patient GlyR autoantibodies. First, patient sera were subjected to untransfected HEK293 cells and cells transfected with the empty pRK5 vector (MOCK control) showing no binding to the cells (Figure 3A). In contrast, patient sera bind to GlyRα1 transfected cells (Figure 3B). An endpoint titration of the sera on transfected HEK293 cells revealed variability in titres between 1:50–1:1000 (Pat1 1:1000; Pat2 1:500; Pat3 1:50 (titration experiment could not be performed due to lack of material); Pat4 1:500, Pat5 1:50; Figure 3B). Interestingly, all five patient sera (Pat1-5), which recognized the glycosylated GlyRα1^WT^, were also able to bind the non-glycosylated GlyRα1^N38Q^ isoform (Figures 4A,B), thus arguing that receptor glycosylation is not essential for GlyR autoantibody binding.

### The non-glycosylated GlyRα1^N38Q^ isoform results in altered functionality of the ion channels

**Figure 5:**
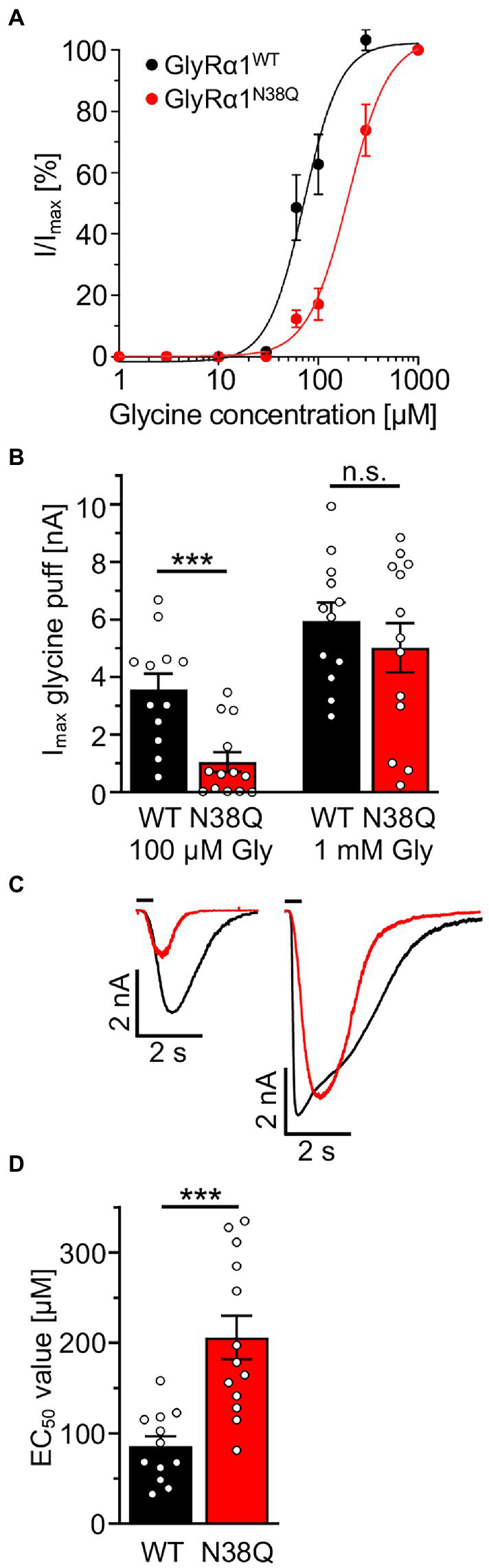
Non-glycosylated GlyRα1 differs in functionality to glycosylated receptors. **(A)** Dose–response curves resulting from electrophysiological recordings of transfected HEK293 cells with GlyRα1^WT^ (black) and GlyRα1^N38Q^ (red). Glycine was applied in a concentration series of 1, 3, 10, 30, 60, 100, 300, 1,000 μM glycine. **(B)** Maximal currents of transfected HEK293 cells upon glycine application of 100 μM (left) or 1 mM glycine (right). **(C)** Representative current traces of GlyRα1^WT^ (black) and GlyRα1^N38Q^ (red) upon application of 100 μM glycine (left traces) and saturating glycine concentration at 1 mM (right traces). **(D)** EC_50_ values determined from dose–response curves of GlyRα1^WT^ (black) and GlyRα1^N38Q^ (red). Data result from three independent experiments (*n* = 3) with 12–13 cells total recorded. Significance values reflect ***p < 0.001.

To investigate whether the GlyRα1^N38Q^ mutant shows functional alterations, we performed electrophysiological recordings. The dose–response curve of GlyRα1^N38Q^ revealed an increased glycine EC_50_ value of 206 μM compared to GlyRα1^WT^ with an EC_50_ of 85 μM (*p* = 0.00029; Figures 5A,D). Comparing the absolute currents of GlyRα1^WT^ and GlyRα1^N38Q^ at a glycine concentration of 100 μM, a significant reduction of the I_max_ values was obvious for the mutant (WT: 3.6 ± 0.5 nA; N38Q: 1.0 ± 0.3 nA; *p* = 0.0006) which was absent at saturating concentrations of glycine (1 mM; GlyRα1^WT^: 6.0 ± 0.6 nA; GlyRα1^N38Q^: 5.0 ± 0.8 nA; *p* = 0.398) (Figures 5B,C). These data indicate a decreased glycine potency of GlyRα1^N38Q^ whereas the efficacy is unaltered.

### Efficient adsorption of GlyRα1 autoantibodies by native GlyRs and non-glycosylated GlyR ECDs purified from *Escherichia coli*

**Figure 6:**
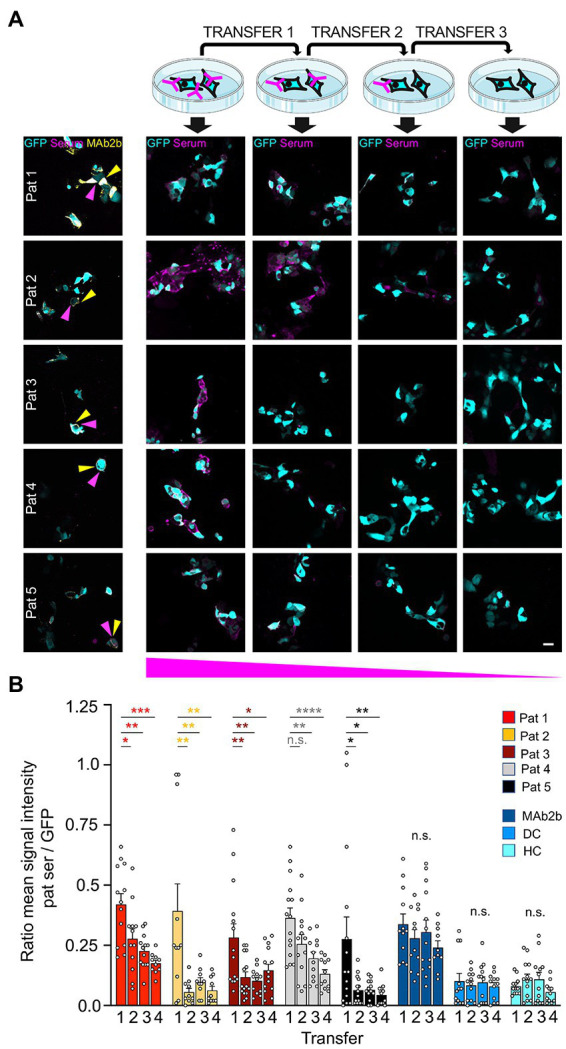
High expression level of GlyRα1 reduce GlyR autoantibodies with high efficiency. **(A)** GlyRα1^WT^ and GFP (cyan) co-transfected HEK293 cells were incubated with patient sera (1:50 in MEM medium with supplements; magenta) and supernatant was transferred to the next dish with transfected cells (scheme at the top). Afterwards, immunocytochemical stainings were performed including co-stainings with MAb2b (left column, yellow). Note the decrease in autoantibody signal the more often the supernatant was transferred (from left to right). **(B)** Quantification of ratio of the mean signal intensity of patient serum signal to mean signal intensity of GFP signal. Calculations of cells incubated with patient sera and controls MAb2b, disease control (DC) and healthy control (HC) are shown. The GFP signal indicated the positively transfected HEK293 cells. Significance values were calculated by comparison of the values from coverslips 1, 2, 3, and 4. Data were derived from four independent experiments (*n* = 4) with a total of 10–15 images being analyzed. Scale bar refers to 20 μm. Significance values reflect *p < 0.05, **p < 0.01, ***p < 0.001, ****p < 0.0001.

As a first life cell-based approach, GlyRα1 transfected HEK293 cells were incubated with patient autoantibodies for 1 h. This approach presumes that GlyR autoantibodies bind to the proposed N-terminal epitope A^29^-G^62^ and are adsorbed by access to the native GlyR ECD. Afterwards, the supernatant was transferred three times to another dish with transfected cells for further incubation. For all patient samples a decrease in signal intensity was seen following two rounds of incubation with transfected cells (Figure 6A; controls shown in Supplementary Figure S1A). The sera of patients 2 and 5 required three rounds of transfer until the adsorption was almost complete (Figure 6A). For verification that autoantibodies are not degraded during experimental procedure, coverslips without cells were used for transfers 1 and 2, and finally incubated with transfected HEK293 cells. Normal binding of autoantibodies to expressed GlyRα1 was detected (Supplementary Figure S1B). Comparing the first and second labelling, the ratio of mean signal intensity of patient sera to the GFP signal was significantly reduced in 4 of 5 patients (Figure 6B). The reduction was significant for all patients when comparing the first to the last labelling (Pat1 *p* = 0.003; Pat2 *p* = 0.020; Pat3 *p* = 0.0214; Pat4 *p* < 0.0001; Pat5 *p* = 0.0085). The commercial GlyRα1-specific antibody MAb2b showed intense labelling even after four rounds of labelling due the high concentration of used antibody (2 μg/ml) (Supplementary Figure S1A). The mean ratio of signal intensities was unaltered between the first and the second as well as first and last labelling when cells were incubated with either MAb2b, HC, or DC (first to last transfer MAb2b *p* = 0.3142, HC *p* = 0.7650, DC *p* = 0.8859; Figure 6B). In sum, autoantibody binding to the native glycosylated GlyR ECD was verified, which consequently represents a suitable tool to efficiently adsorb autoantibodies against the GlyR from patient serum samples.

**Figure 7:**
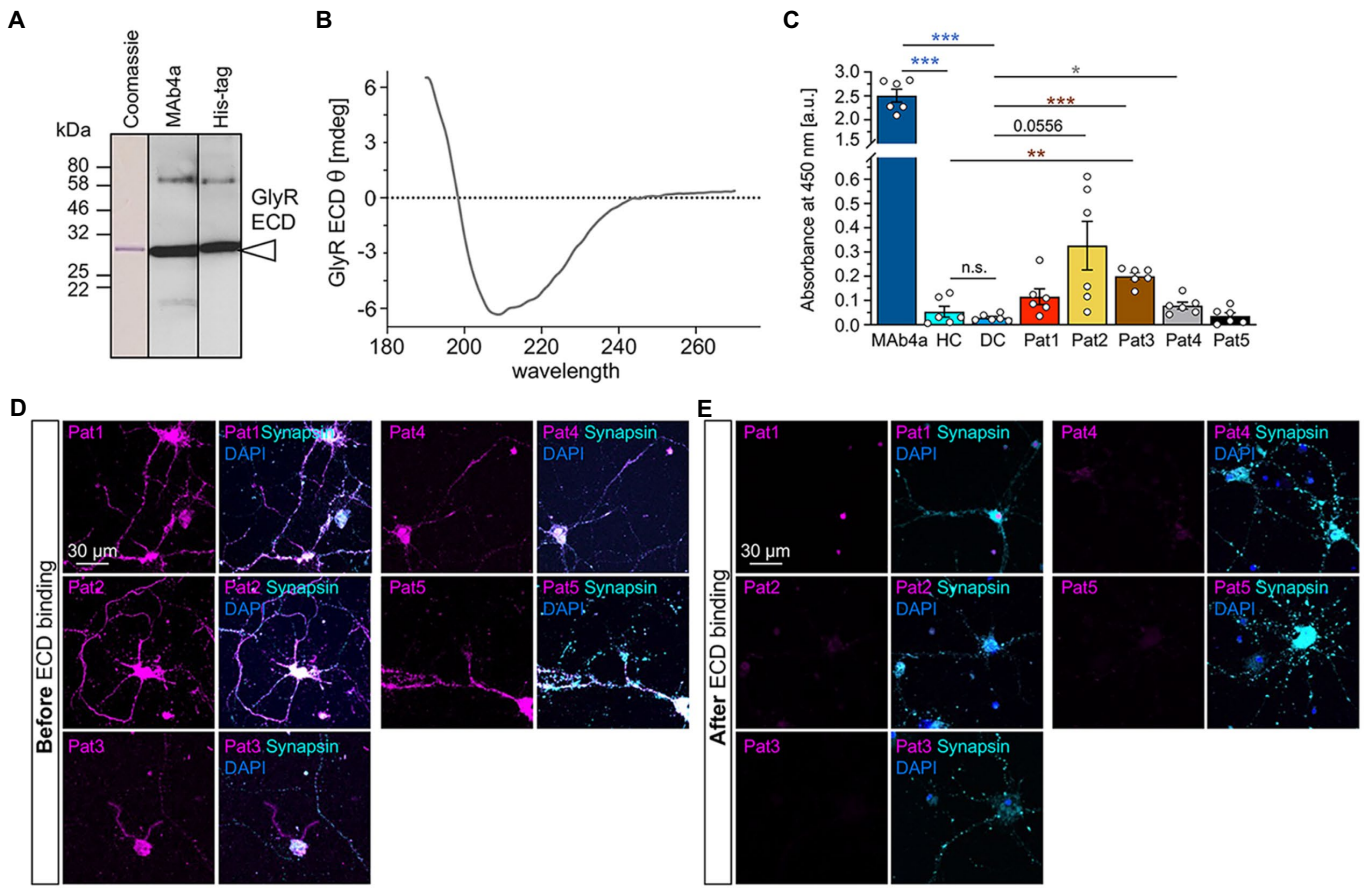
Patient autoantibody binding to the non-glycosylated GlyRα1 ECD prevents subsequent binding to endogenous GlyRs expressed in spinal cord neurons. **(A)** Detection of GlyRα1 ECD construct using Western Blot analysis. Coomassie stain (left) as well as blot stained with MAb4a (middle) or anti-his-tag antibody (right). **(B)** CD spectrum of GlyR ECD after refolding. The spectrum is baseline corrected and represent the average of 10 individual scans. **(C)** Analysis of absorbance values recorded from GlyRα1 ECD coated ELISA plates that were incubated with patient sera (Pat1-5), MAb4a, healthy control (HC) and disease control (DC). Number of measured data were *n* = 6 from 2 independent experiments. All significance values were calculated with unpaired *t* tests followed by a Holms-Sidaks correction for multiple samples. **(D)** Immunocytochemical stainings of cultivated motoneurons (DIV14) with patient serum (magenta) which were performed before autoantibody binding to GlyR ECD. As control stainings, synapsin (cyan) and DAPI (blue) are included. **(E)** After patient autoantibodies were neutralized with the GlyRα1 ECD coated on ELISA plates, the suspension was transferred to cultivated motoneurons (magenta) and stained together with synapsin (cyan) and DAPI (blue). Significance values reflect *p < 0.05, **p < 0.01, ***p < 0.001.

We determined that GlyR glycosylation is not essential for autoantibody binding (Figure 4). Therefore, a GlyRα1 ECD expressed, purified, and re-folded from *E. coli* (Breitinger et al., 2004) may serve as a valuable tool for GlyR autoantibody screening due to efficient binding of GlyR autoantibodies from patient sera. *E. coli* cells are unable to generate glycosylated protein, therefore the verification of GlyR autoantibody binding to non-glycosylated GlyRs was a prerequisite for the use of GlyR ECDs as tool for autoantibody detection. The GlyRα ECD constructs contain a 6xHis-tag and harbours the ligand binding and receptor assembly domains. Purity and folding of the GlyRα ECD protein (29 kDa, dimer at 58 kDa) was verified with Coomassie stain and Western blot using MAb4a or an anti-His-tag antibody as well as CD spectroscopy (Figures 7A,B).

**Figure 8:**
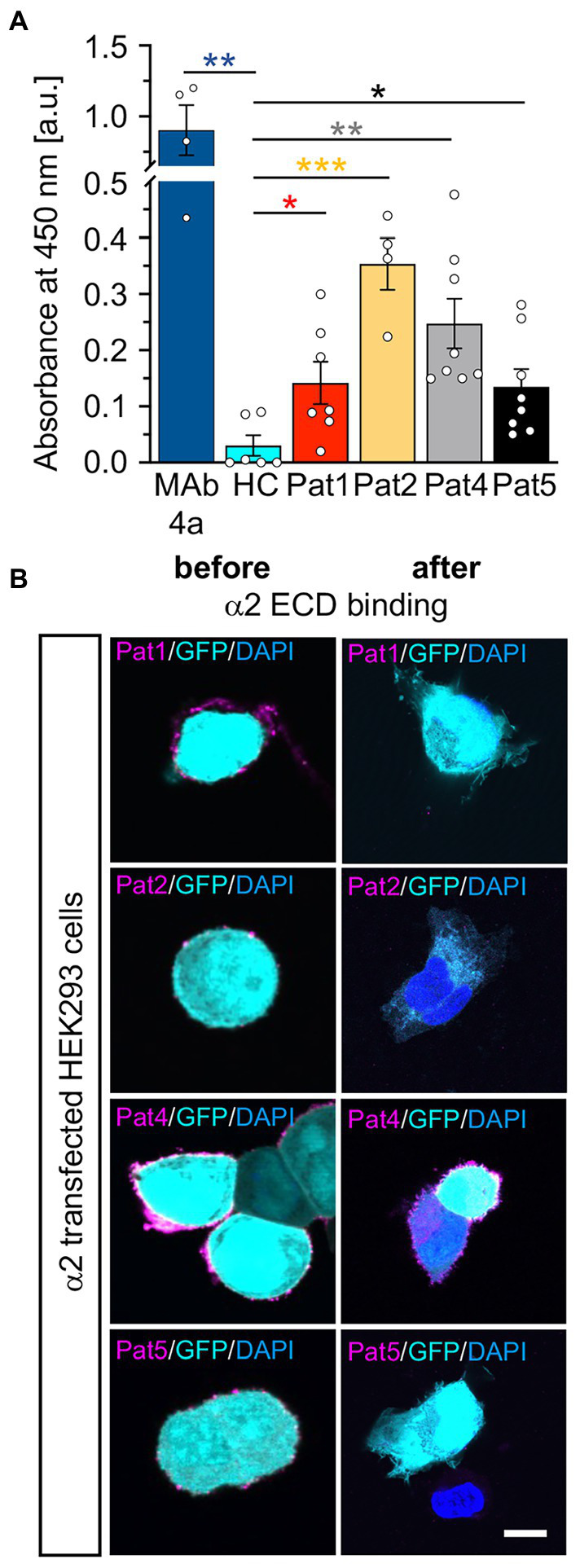
Patient autoantibodies are also efficiently targeting GlyRα2 ECDs. **(A)** Absorbance values detected from samples of patient sera (Pat1, 2, 4, 5; serum of Pat3 was not tested due to limited amount of sample; value of p for Pat1 *p* = 0.0457; Pat2 *p* = 0.0005; Pat4 *p* = 0.0048, Pat5 *p* = 0.0457; MAb4a *p* = 0.0012 always compared to HC) incubated with GlyRα2 ECD coated ELISA plates. Data were obtained from *n* = 4–8 data points measured in two independent experiments. Significances were calculated with a *t* test with Holms-Šidáks correction for multiple samples. **(B)** GlyRα2 transfected HEK293 cells were stained using Pat1, 2, 4, 5 sera or supernatants following incubation on GlyRα2 ECD coated ELISA plates (α2 magenta; GFP cyan, and DAPI blue). Scale bar refers to 10 μm. Significance values reflect *p < 0.05, **p < 0.01, **p < 0.001.

Following coating of ELISA plates with GlyRα1 ECD, the patient autoantibody containing samples were incubated for 1 h with coated wells and transferred twice. The binding of autoantibodies to the GlyRα1 ECD in the well plate was analysed by determining the absorbance at 450 nm (Figure 7C). MAb4a showed the highest absorbance and served as positive control. Patient sera 1–4 showed increased absorbance values compared to healthy and disease control (compared to DC: *p*-values Pat1 *p* = 0.0757, Pat2 *p* = 0.0556, Pat3 *p* = 0.0007, Pat4 *p* = 0.0427, Pat5 *p* = 0.639), indicating that patient autoantibodies bound to the purified non-glycosylated GlyRα1 ECD from *E. coli*. To control for efficient patient autoantibody adsorption, the ELISA supernatants were subjected to primary motoneurons where GlyRs are highly abundant and to GlyRα1 transfected HEK293 cells. In motoneurons, GlyR-labelling of patient autoantibodies was largely reduced whereas the synapsin signal was constant independent of the ELISA adsorption of autoantibodies (Figures 7D,E). The same autoantibody signal reduction was identified when the suspension was transferred to GlyRα1 transfected HEK293 cells (Supplementary Figure S2). It has been shown that GlyR autoantibodies do not only target the α1 subunit but also the α2 and α3 subunits (Carvajal-Gonzalez et al., 2014). Using a similar approach with the purified ECD of the GlyRα2 subunit in an ELISA, patient autoantibodies against the α2 subunit were also efficiently bound and thus verified by two independent measures using a cell-based experiment and an ELISA-based assay (Figures 8A,B). Pat3 was excluded from this experiment due to a lack of sufficient serum material. Interestingly, the serum of Pat4 showed still binding to with GlyRα2 transfected HEK293 cells in the cell-based assay following the ELISA. This result demonstrates that GlyR autoantibodies remained in the diluted serum sample which further suggests that the serum titre of GlyR autoantibodies from Pat4 might be higher than that of the other samples or an inter-subunit epitope between two adjacent GlyR subunits exists which can only be presented by native pentameric GlyRα2 receptors but not by the GlyRα2 ECD. The titre of Pat4 was with 1:500 higher than for Pat5 but similar to Pat2 and Pat1 (Figure 3B).

In sum, the non-glycosylated GlyRα1 did not exhibit large structural alterations, was to a significant degree transported to the cell surface and reveals an increased glycine potency while glycine efficacy was unaffected. As glycosylation is not a prerequisite for GlyR autoantibody binding, GlyR ECD domains purified from *E. coli* represent a valuable tool to screen for patient autoantibodies with high efficiency in addition to the so far used cell-based assays on living cells.

## Discussion

The present study expands the current knowledge on the pathophysiology of GlyR autoantibodies by demonstrating that autoantibody binding is independent of receptor glycosylation. Furthermore, GlyR autoantibodies can be efficiently bound by the presentation of purified non-glycosylated GlyR ECD harboring the autoantibody epitope suitable for ELISA-based detection.

GlyR autoantibodies are associated with SPS (Dalmau et al., 2017). Previously, receptor internalization and complement activation, functional alterations of the targeted inhibitory GlyRs upon autoantibody binding and the definition of a common epitope of GlyR autoantibody binding ^29^A-^62^G were shown to underlie the pathology (Carvajal-Gonzalez et al., 2014; Crisp et al., 2019; Rauschenberger et al., 2020). The pathogenicity of GlyR autoantibodies and thus the autoimmune etiology of the disease was confirmed by passive transfer of patient serum to zebrafish larvae, that yielded an abnormal escape response – a brainstem reflex that corresponds to the exaggerated startle of afflicted patients (Rauschenberger et al., 2020).

The glycosylation site of the GlyR is in close proximity to the identified GlyR autoantibody epitope. Glycosylation occurs at position Asn38 which is conserved among the GlyRα subunits α1-3 as well as in the β subunit (Pult et al., 2011; Schaefer et al., 2018). Glycosylation of proteins targeted by autoantibodies has been discussed for the excitatory NMDARs in NMDAR encephalitis but also for adhesion proteins, e.g., Caspr2 and Contactin-1 in autoimmune-mediated neuropathies (Gleichman et al., 2012; Labasque et al., 2014; Olsen et al., 2015). Interestingly, for the NMDARs it was demonstrated that not glycosylation itself but the structural environment of the glycosylation site is important for autoantibody binding (Gleichman et al., 2012; Castillo-Gomez et al., 2017). Therefore, we concentrated first on the structural surrounding of the GlyR glycosylation site based on the high-resolution X-ray structure of the GlyRα3 homopentamer (Huang et al., 2015). The modeling analysis showed a loss of hydrogen bond between the glycan and Asn31 residing in the neighboring loop, yet the mutant version (N38Q) possibly retained the interaction with the loop by interacting with the main chain carboxylate of Pro36. Hence, we would not expect changes in autoantibody binding to non-glycosylated GlyR protein. Receptor glycosylation, however, was described as a prerequisite for receptor trafficking, especially for endoplasmic reticulum exit of assembled GlyRs (Griffon et al., 1999). Protein transport of non-glycosylated GlyRs to the cellular surface has however been observed in previous reports although to a minor extent compared to glycosylated receptor protein (Schaefer et al., 2015). We quantified the fractions of membrane bound non-glycosylated GlyRα1^N38Q^ and found reduced receptor numbers (65%) at the cellular surface. From genetic GlyR variants it is known that a reduced surface expression level of 53% still exhibit normal GlyR functionality (Atak et al., 2015; Schaefer et al., 2015).

Electrophysiological recordings of GlyRα1^N38Q^ revealed an increased EC_50_ value compared to GlyRα1^WT^ indicating a reduced glycine potency of the mutant. Comparable reductions of glycine potency have also been documented for another GlyR mutant next to the glycosylation site namely A52S (Ryan et al., 1994; Saul et al., 1994). In addition, binding of GlyR autoantibodies to the N-terminal region neighbouring the glycosylation site also affected glycine potency (Rauschenberger et al., 2020). Maximal currents upon application of saturating glycine concentrations (100 μM) were unchanged for the non-glycosylated GlyRα1^N38Q^ variant arguing for no change of the neurotransmitter efficacy. These results are in line with cryo-electron microscopy (EM) data demonstrating that the region around the glycosylation site is involved in channel transitions between the open and closed states rather than acting as target for the agonist glycine (Du et al., 2015). Moreover, recent studies on GlyR stoichiometry using cryo-EM showed that N-linked glycosylation sites at position N38 in GlyRα subunits facing away from the ion channel vestibule, while the corresponding N-linked glycosylation site in GlyRβ is turning into the vestibule and hence represents an essential component for heteromeric assemblies of αβ heteromers (Yu et al., 2021). Our data imply that the removal of glycosylation at asparagine 38 in GlyRα1 led to lower receptor expression at the cell surface. Although less expressed, patient autoantibodies are still able to bind the mutant GlyRα1^N38Q^. In sum, GlyR glycosylation is not a prerequisite for GlyR autoantibody binding, similar to observations for other autoantibodies against ion channels such as the anti-NMDAR autoantibodies in patients with encephalitis (Gleichman et al., 2012; Castillo-Gomez et al., 2017).

As the common GlyRα1 autoantibody epitope is localized in the N-terminal part of the ECD between residues ^29^A-^62^G, the purified and refolded GlyRα1 ECD was efficient to bind autoantibodies from patient sera. Similarly, patient sera positive for GlyRα2 showed binding to the α2 ECD. This was demonstrated by binding of GlyR autoantibodies to GlyRα1/α2 ECDs coated to ELISA plates followed by binding assays to motoneurons or transfected HEK293 cells to show specificity of the assay. Therefore, the successful investigation of using GlyR domains or even full-length receptors as additional fast option for screening of patient sera for GlyR autoantibodies offers an additional tool besides testing binding to living cells (Vincent et al., 2012). Treatment of SPS patients includes plasma exchange, glucocorticosteroid application, and immunosuppression to reduce the autoantibody titre in patients’ blood (Nydegger and Sturzenegger, 2001; Shariatmadar and Noto, 2001; Hutchinson et al., 2008;Pagano et al., 2014; Albahra et al., 2019). Plasma exchange is well tolerated and 78% of the patients improve at least to some extent from this therapy (Pagano et al., 2014; Albahra et al., 2019). However, litres of blood have to be exchanged to remove 1–2 g of pathogenic IgG during plasmapheresis while at the same time eliminating 150 g of healthy protein simultaneously (Pineda, 1999). For developing better and specific antibody-selective immunotherapies, the identification of patient-derived monoclonal antibodies and the characterization of their binding properties to further investigate the disease pathologies is an essential step (Kreye et al., 2021). The binding pattern of patient-derived monoclonal antibodies can be studied using purified full-length receptors or domains in distinct conformations bound to ELISA plates followed by structural analysis, e.g., single particle cryo-EM of combined receptor and autoantibody proteins and functional analyses (Chou et al., 2022; Noviello et al., 2022; Tajima et al., 2022). Such combined approaches will provide detailed binding pattern pictures of patient-derived autoantibodies opening novel windows for clinical treatment using antibody depletion strategies.

In sum, we could show that the non-glycosylation mutant GlyRα1^N38Q^ is expressed at the cell surface and is functionally active. Receptor glycosylation is not required for binding of patient GlyR autoantibodies. GlyR autoantibodies were efficiently bound by offering the GlyRα ECD as a target. ELISA plates coated with GlyR ECD thus provide another possibility to investigate autoantibody titers from patient samples with high precision besides cell-based assays. In addition to purified domains of receptor proteins, the increasing knowledge from cryo-EM structures using various approaches, e.g., large scale purification of folded full-length ion channels from insect cells represents a future direction to translate findings from basic structural biology towards improvements of clinical diagnosis.

## Data availability statement

The original contributions presented in the study are included in the article/supplementary material, further inquiries can be directed to the corresponding author/s.

## Ethics statement

Written informed consent was obtained from the individual(s) for the publication of any potentially identifiable images or data included in this article.

## Author contributions

VR, IP, and A-LE performed immunocytochemical stainings, western blots, biotinylation assays, electrophysiological recordings, and adsorption experiments with HEK293 cells and ELISA. VH did the ELISA tests followed by staining of cultured motoneurons. Statistical analyses were done by VR and IP. Structural investigations were provided by VK and HS. ECDs were produced by CK. Patient analyses, description, and sera were provided by H-MM, ET, CS, KD, and CG. Project design and development was done by CK, H-MM, CS, CG, KD, ET, and CV. CV wrote the manuscript with contribution from all coauthors. All authors contributed to the article and approved the submitted version.

## Funding

This work was supported by Deutsche Forschungsgemeinschaft SO328/9-1 (CS) and VI586/8-1 (CV), GE2519/8-1 (CG), Research unit SYNABS FOR3004. VR, IP and A-LE are supported by the GSLS Würzburg, Germany.

## Conflict of interest

The authors declare that the research was conducted in the absence of any commercial or financial relationships that could be construed as a potential conflict of interest.

## Publisher’s note

All claims expressed in this article are solely those of the authors and do not necessarily represent those of their affiliated organizations, or those of the publisher, the editors and the reviewers. Any product that may be evaluated in this article, or claim that may be made by its manufacturer, is not guaranteed or endorsed by the publisher.

## Footnotes

^1^ www.pymol.org
