# Supplementary material for: Glycine receptor autoantibody binding to the extracellular domain is independent from receptor glycosylation
Source: Front Mol Neurosci. 2023 Feb 13;16:1089101. doi: 10.3389/fnmol.2023.1089101 (PMC9969106; doi:10.3389/fnmol.2023.1089101)
Supplement: Supplementary file 1 [file Data_Sheet_1.PDF]

# *Supplementary Material*

Vera Rauschenberger et al.

Glycine receptor autoantibody binding to the extracellular domain is independent from receptor glycosylation

## 1 Supplementary Figures and Tables

### Supplementary Figure 1

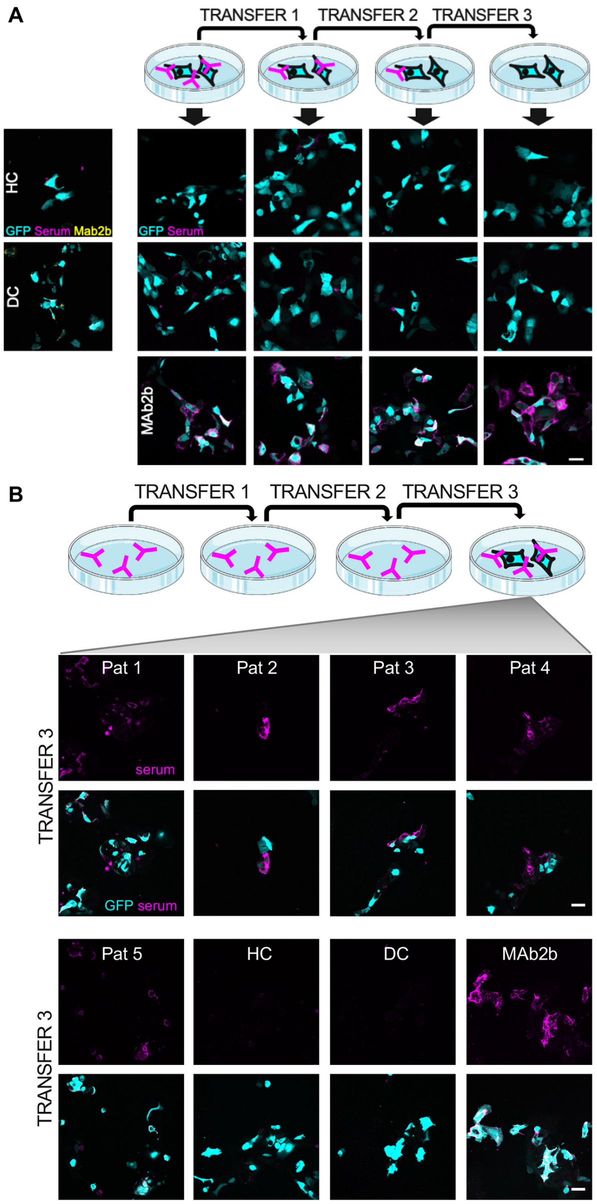

**Supplementary Fig. 1.** Neutralization controls and test for autoantibody degradation. (A) HEK293 cells transfected with GlyRα1$^{WT}$ and GFP (cyan) were incubated with healthy control (HC), disease control (DC) (1:50 in MEM medium with supplements) or MAb2b antibody (1:500) and supernatant was 3x transferred to the fresh transfected cells (scheme at the top). Immunocytochemical stainings including co-stainings with MAb2b (left column, yellow) were conducted. (B) Patient autoantibodies were 3x incubated in dishes without HEK293 cells for 1 h each. After, the solution was transferred to GlyRα1$^{WT}$ and GFP (cyan) co-transfected HEK293 cells followed by immunocytochemical stainings. Scale bar refers to 20 µm.

**Supplementary Figure 2**

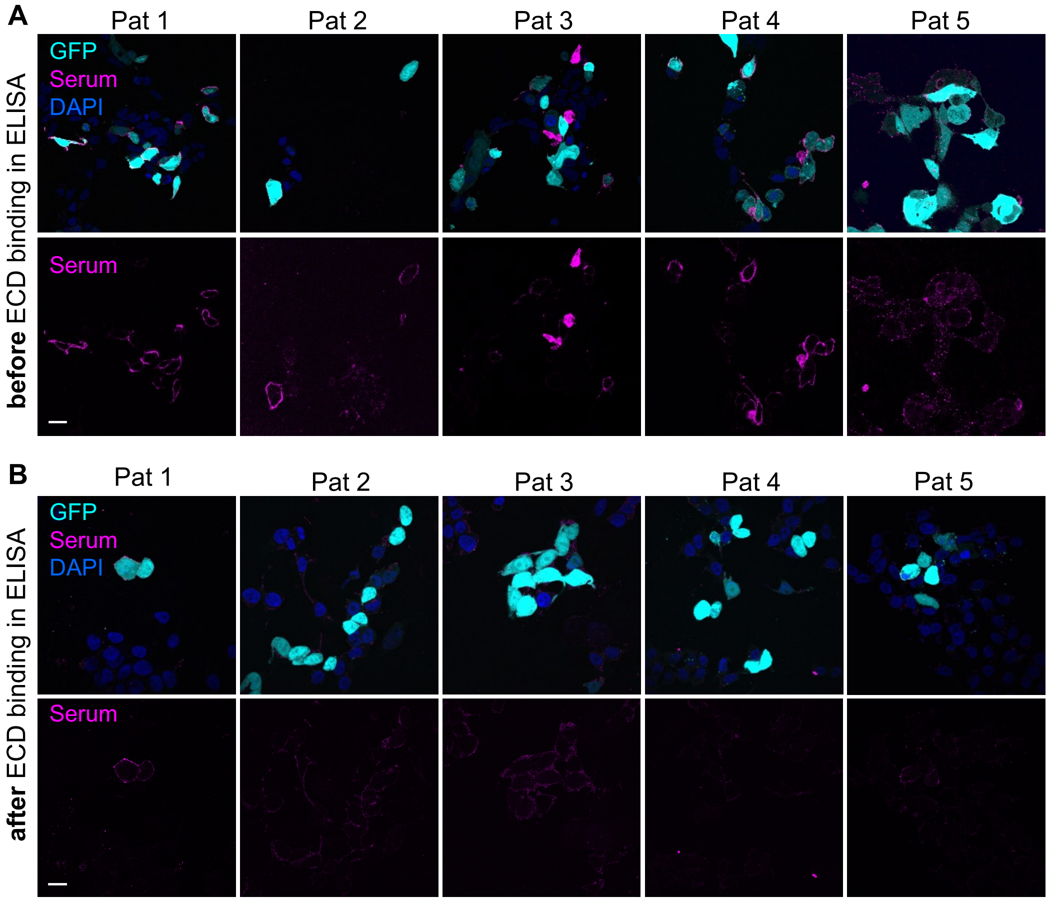


**Supplementary Fig. 2.** Preincubation of GlyR autoantibodies with the purified GlyRα1 ECD prevents autoantibody binding to overexpressed GlyRs in HEK293 cells.

(A) Immunocytochemical stainings of GlyRα1 and GFP (cyan) co-transfected HEK293 cells with patient serum (magenta) which were performed before autoantibody binding to GlyR ECD. DAPI staining (blue) was included. (B) After patient autoantibodies were neutralized with the GlyRα1 ECD coated on ELISA plates, the suspension (magenta) was transferred to transfected HEK293 cells and stained together with DAPI (blue). Scale bar refers to 20 µm.
